# Light-Cured Dental Fillings Containing Quinoline and Quinoxaline Derivatives: The Influence of Sorption and Solubility on Color Change—Part III

**DOI:** 10.3390/ijms26199537

**Published:** 2025-09-29

**Authors:** Ilona Pyszka, Dawid Bereźnicki, Beata Jędrzejewska

**Affiliations:** Faculty of Chemical Technology and Engineering, Bydgoszcz University of Science and Technology, Seminaryjna 3, 85-326 Bydgoszcz, Poland

**Keywords:** quinoline and quinoxaline derivatives, photopolymerization, light-cured composites, sorption, solubility, color change

## Abstract

Light-cured dental fillings play a significant role in modern dentistry due to their aesthetics, durability, and ease of application. However, research is still being carried out to improve their mechanical properties, biocompatibility, and wear resistance by modifying their composition. Therefore, the aim of this study was to evaluate the sorption properties, solubility, and color stability of newly developed dental composites containing quinoline and quinoxaline derivatives. A total of 162 samples were prepared by mixing organic and inorganic phases. For 144 of these, color changes were assessed after conditioning in solutions simulating the oral environment, and for 18, sorption and solubility analysis were performed in distilled water according to ISO 4049. The results showed that sorption and solubility increased with increasing conditioning time. Composites characterized by higher matrix hydrophilicity showed both greater sorption and solubility as well as more pronounced color changes (Δ*E* > 6). In contrast, materials with lower sorption and solubility were characterized by greater color stability (Δ*E* < 5). The results indicate a significant impact of the hydrophilic properties of the resin matrix on the aesthetic durability of dental restorations. The data obtained can provide a basis for the appropriate design of biofunctional materials with increased resistance to degradation and discoloration.

## 1. Introduction

Polymer composite materials are the basis of modern conservative and aesthetic dentistry. Their versatile applications—from the reconstruction of cavities in anterior and posterior teeth, through temporary fillings and sealing fissures, to the cementation of inlays and prosthetic restorations—have contributed to their widespread acceptance in clinical practice worldwide [1]. These multi-component systems primarily consist of an organic matrix (methacrylate resins: most commonly Bis-GMA, UDMA, or TEGDMA) and an inorganic filler phase (glass, silica, or ceramic particles), as well as polymerization initiators (e.g., camphorquinone), stabilizers, dyes, and other additives that modify physicochemical properties. The final functional parameters of the dental material depend on both its chemical composition and the method and efficiency of the polymerization process [2,3].

In terms of chemical composition, one of the key elements influencing the properties of these materials is the photoinitiator—a chemical substance responsible for initiating the polymerization reaction, which allows the filling to harden when exposed to light. Choosing the right type of photoinitiator directly impacts the quality, durability, and aesthetics of dental fillings. A properly selected photoinitiator ensures effective and rapid hardening of the composite material, which translates into its mechanical strength, abrasion resistance, and aesthetic stability. The field of photoinitiator systems is still developing rapidly. Numerous studies have been reported in the literature on composites containing traditional photoinitiators, including camphorquinone and its derivatives [4,5], as well as phenanthrenequinone [6], benzophenone [7], 1-phenyl-1,2-propanedione [8], trimethylbenzoyldiphenylphosphine oxide [9,10], and benzoyl peroxide [11]. It has been shown that even such a small component of dental composites has a huge impact on their biomechanical and chemical properties.

Finding a compromise between appropriate mechanical properties and the aesthetic appearance of the filling, however, remains a major challenge [12]. For example, trimethylbenzoyldiphenylphosphine oxide has been shown to accelerate the polymerization, which shortens the material’s application time, but causes higher shrinkage stresses and is characterized by a lower depth of cure. 1-Phenyl-1,2-propanedione, on the other hand, reacts more slowly than camphorquinone [13], leading to a lower polymerization rate and reduced crosslinking density. Consequently, the material becomes more susceptible to a solvent and enzymatic attack and therefore exhibits poorer mechanical properties. When phenanthrenequinone was used, composites were obtained whose color stability was lower than in systems containing camphorquinone [6]. These limitations have led to the search for new photoinitiating systems that could provide a better balance between the aesthetics, stability, and mechanical properties of dental materials.

Oral fillings are exposed to many physicochemical factors, such as varying pH, temperature fluctuations, mechanical abrasion, and enzymatic action. Furthermore, hygiene products—especially bleaching agents containing hydrogen peroxide—can degrade the matrix, resulting in microcracks, loss of gloss, or the release of uncured components [14,15,16]. As demonstrated by Pecho et al. [17], long-term exposure to bleaching agents also significantly reduces the mechanical strength of composites.

One of the key factors influencing the durability of a filling is its mass stability. This stability is influenced not only by the material’s properties but also by the characteristics of the liquid with which it comes into contact. It has been proven that the solubility of polymeric materials increases in acidic or alcohol-containing solutions compared to pure water [18,19]. Acidic solutions can lead to degradation of the filler surface, which results in loss of composite mass [20]. However, the oral cavity is dominated by aqueous solutions of various compositions and pH, which, by diffusion through the material structure, can induce a number of physical and chemical changes. These processes may include, among others, an increase in the material’s volume, swelling, softening, oxidation, hydrolysis, or a change in elasticity [18].

The degree of environmental impact on a material’s mass depends largely on the hydrophilicity of its polymer matrix. The greater its susceptibility to interactions with water, the stronger its tendency to sorption of liquids from the environment [4,18,19,20,21,22,23]. Hydrophilic components have the ability to form hydrogen and polar bonds, which promote water retention in the material structure [18,20]. Important factors influencing this phenomenon include the material’s porosity and the degree of polymer cross-linking—liquids penetrate more easily through more porous structures with lower bond density. Materials with larger intermolecular spaces are particularly susceptible to this. In clinical practice, to limit unfavorable sorption and solubility processes, the proportion of inorganic filler is increased, as it is the organic part of the composite that is primarily responsible for these phenomena [4,18,24,25].

From the aesthetic perspective of dental restorations, the material’s resistance to discoloration [26,27] is particularly important. This phenomenon depends on factors such as the material’s ability to absorb dyes from the environment (e.g., from coffee, tea, or colored drinks), the hydrophilicity of the resin matrix, the type and size of the filler particles, and the surface roughness. Studies conducted on a group of commercial nanohybrid composites (RBCs) have demonstrated a linear relationship between color change and water solubility [28,29,30]. Furthermore, the exposure time in a given medium positively correlates with the magnitude of color change—the longer the exposure, the greater the color deviation from the original [31,32].

Current trends in the development of composite materials focus on improving aesthetics, increasing mechanical strength, and reducing negative biological effects. Despite the widespread use of methacrylate resins, their limitations—such as sensitivity to discoloration, release of monomers, or susceptibility to hydrolytic degradation—remain a clinical challenge [2,3]. In response to these problems, new material strategies are being developed, such as modifications to the resin structure and the use of innovative compounds with bioactive and stabilizing properties. Of particular interest are derivatives of heterocyclic compounds, including quinoline and quinoxaline, which exhibit a broad spectrum of biological activity—antibacterial, antiviral, anticancer, antimalarial, and antifungal [33,34,35,36,37]. Their use as photoinitiating components in a polymer matrix can not only improve aesthetics and resistance to discoloration but also limit the development of biofilm on the surface of fillings [38].

In this work, which is a continuation of previous studies (Parts I and II) [38,39], an in-depth characterization of a newly developed photocomposite containing selected quinoline and quinoxaline derivatives with high color stability and reduced solubility was carried out. The focus was on two key performance parameters, water sorption and solubility, and their relationship to color stability in conditions similar to those found in the oral cavity. Both aspects are fundamental to long-term aesthetics. The obtained results will allow for a preliminary assessment of the material’s suitability for use in aesthetic and conservative dentistry.

## 2. Results and Discussion

This chapter discusses the study of a newly developed composite that combines optimal color stability with resistance to the conditions found in the oral cavity, where the presence of various chemicals can affect the properties of dental materials. The tested dental compositions contain quinoline and quinoxaline derivatives as photoinitiators, (phenylthio)acetic acid as coinitiators, trimethylolpropane triacrylate as a monomer, and dental glass as a filler. Camphorquinone, ethyl 4-dimethylaminobenzoate, bisphenol A glycerolate dimethacrylate, and dental glass were used to prepare a comparative composite. The structures of the compounds are presented in Table 1.

### 2.1. Spectrophotometric Color Analysis

The aesthetics of dental fillings largely depend on their color stability, which is influenced by the ability of composite materials to absorb pigments and dyes from the surrounding environment. The properties of the matrix, including its hydrophilicity, and filler parameters, such as particle size, are crucial. Furthermore, surface roughness is a significant factor influencing the material’s susceptibility to discoloration. Studies conducted on selected commercial composite resins indicate a linear relationship between color changes and the material’s solubility in an aqueous environment [21,28,30,40,41]. In addition, the extent of observed discoloration tends to increase with increasing exposure time to coloring solutions [31,32,42].

**Figure 1:**
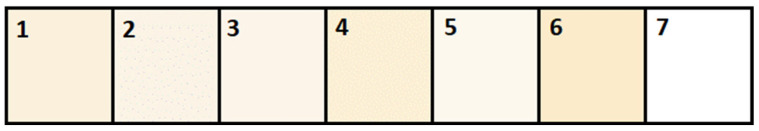
Initial color of tested materials: 1—DQ1-PhTAA, 2—DQ2-PhTAA, 3—DQ3-PhTAA, 4—DQ4-PhTAA, 5—DQ5-PhTAA, 6—CQ-EDMAB, 7—reference.

Figure 1 shows the initial color of the tested materials, obtained based on the average values of the *L*, *a* and *b* coordinates in the CIE Lab color space, marked on the surface of the tested material samples before the conditioning process. In this three-dimensional color space model, the *L* coordinate defines the lightness of a color, from 0 (absolute black) to 100 (diffuse white), regardless of its hue or saturation. The *a* coordinate represents the color’s position between the green and red poles (from −120 to +120). The *b* coordinate, on the other hand, defines the color’s position between the blue and yellow poles (from −120 to +120).

**Figure 2:**
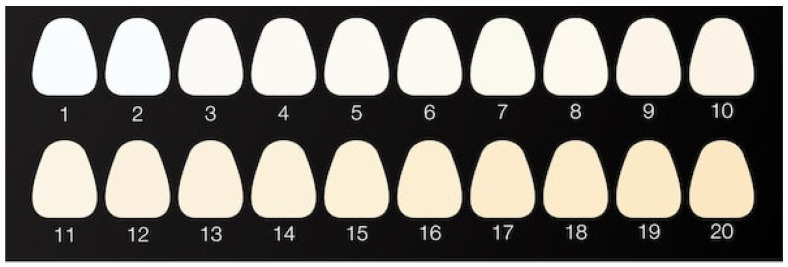
Teeth whiteness chart.

Figure 2 shows the tooth whiteness chart used in this study. The results were analyzed based on the reference, which was sample 1 from the chart (sample 7 in Figure 1). Reference parameters were *L* = 98.93, *a* = −0.28 and *b* = 0.54.

First, the initial color of the analyzed samples was compared with the tooth whiteness sample. Based on the measured values of the *L*, *a*, and *b* parameters, the initial color difference (Δ*E*) of the tested materials was calculated relative to the reference (Table 2).

The results showed that in all studied cases, statistically significant color differences compared to the color of ideal white coincided with the point where the clinically acceptable Δ*E* limit is exceeded (Δ*E* > 3.3). However, it should be noted that perfectly white enamel color occurs extremely rarely, as the shade of enamel is an individual characteristic of each patient.

Enamel characteristics indicate that brightness (*L*) depends primarily on enamel thickness—the thicker the layer, the higher the *L* value. Shades *a* (green-red) and *b* (blue-yellow) are influenced by the structure and composition of the enamel and the color of the underlying dentin. Therefore, the natural color of enamel has a slightly bluish-gray tone, while the warm, yellowish tint is largely due to the dentin showing through [43]. Selecting the appropriate shade of fillings and crowns is therefore crucial to achieving a harmonious, natural aesthetic effect.

**Figure 3:**
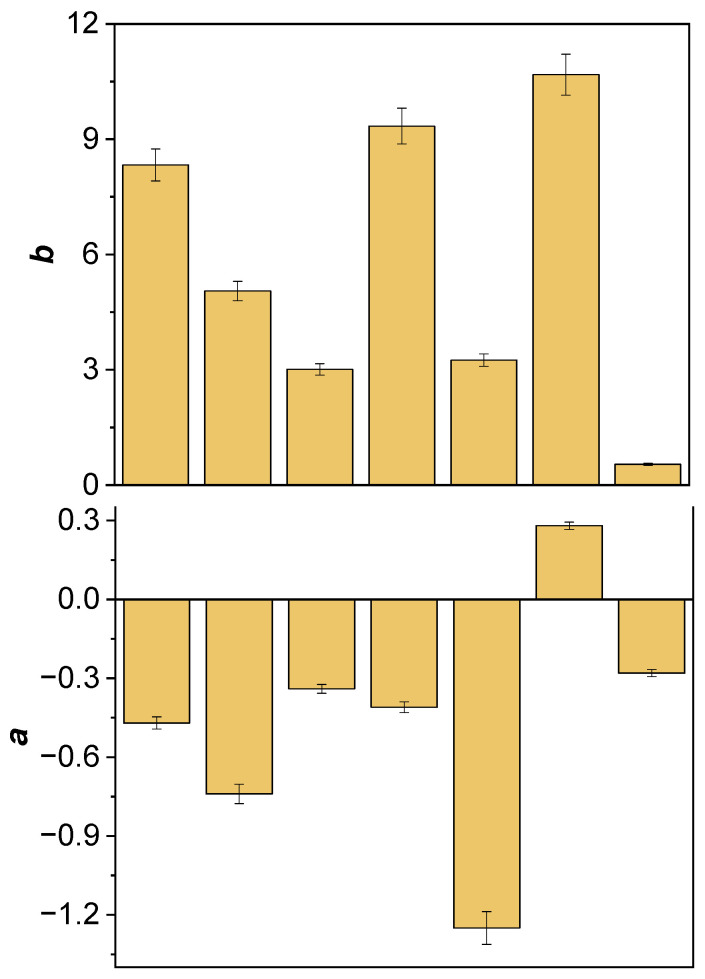

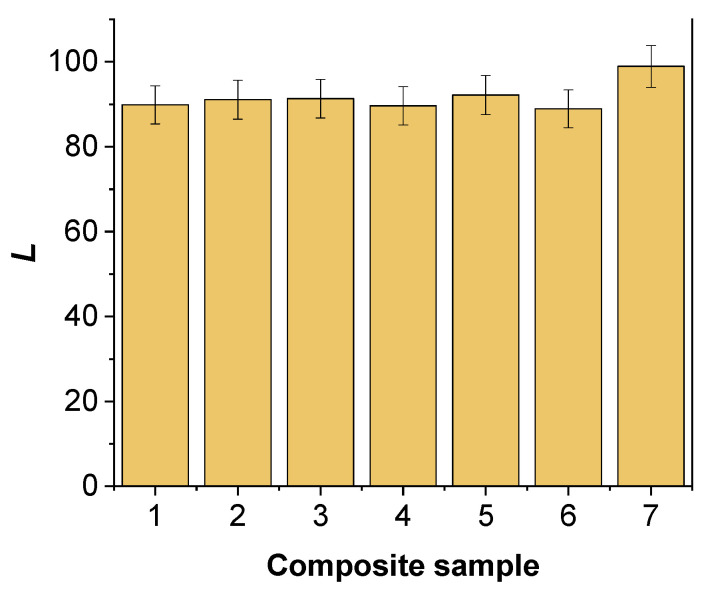
Initial average value of the parameter *L* (brightness) and parameter *a* and *b* coordinates of the tested materials: 1—DQ1-PhTAA, 2—DQ2-PhTAA, 3—DQ3-PhTAA, 4—DQ4-PhTAA, 5—DQ5-PhTAA, 6—CQ-EDMAB, 7—reference sample.

Among the analyzed samples, the largest color differences from ideal white were observed in commercial composite sample 6 (Δ*E* = 14.57), which may indicate increased hydrophilicity of the matrix. The smallest color differences were observed in samples 5 (Δ*E* = 7.02) and 3 (Δ*E* = 7.70), likely due to their lower surface roughness. Figure 3 present the initial average values of the *L*, *a*, and *b* coordinates for the tested materials in the CIE Lab color space.

The differences in the *L* parameter values (Figure 3) clearly demonstrate that individual composite samples differ in brightness. Higher *L* values correlate with a brighter visual perception of the sample, which may be due to both the properties of the dyes used as photoinitiators in the organic matrix and the microscopic surface topography (e.g., roughness, porosity). Data analysis indicates that composites containing DQ3-PhTAA (sample 3) and DQ5-PhTAA (sample 5) photoinitiating systems achieved the highest *L* values, similar to the reference, making them the materials with the highest lightness in the tested group. In contrast, the commercial composite (sample 6) had the lowest *L* value and therefore the darkest color. Such a wide range of brightness suggests that the selection and concentration of photoinitiators, as well as appropriate treatment of the sample surface, are crucial for obtaining the optimal aesthetic effect in the final dental material.

Figure 3 also illustrates the initial average values of the *a* parameter in the CIE Lab color space for the tested materials. The *a* parameter controls the chromatic component along the green-red axis—negative values indicate a color shift toward green, while positive values indicate a color shift toward red. Data analysis shows that all tested samples, except the commercial reference material (sample 6), are characterized by negative values of the *a* coordinate. This distribution indicates that most of the tested samples have a natural, greenish tint, while the reference material has a more pronounced red tint, typical of commercial standards.

The initial mean values of the *b* parameter, which corresponds to the color shift along the blue-yellow axis, are also illustrated in Figure 3. Positive values of the *b* coordinate, observed in all samples, indicate a predominance of yellow in their initial color. Variation in the *b* parameter between individual materials is related to differences in the absorption maximum position of the photoinitiators used (DQ1–DQ5, CQ) in the organic matrix. The electronic absorption spectra of these compounds, located in the violet-blue region (400–480 nm) [38], determine the complementary color perceived by the human eye as a green-yellow or yellow hue. Photoinitiators DQ1 and DQ4 exhibit maximum absorption at 432 nm and 408 nm, respectively, resulting in the most saturated yellow hue in the study group. Camphorquinone (CQ), on the other hand, absorbs visible light at λ_max_ = 472 nm, thanks to which both it and the composite containing it present the most intense yellow color (Figure 3, sample 6). The higher the *b* parameter value, the more “warm” and yellowish the composite hue. This characteristic is crucial when selecting raw materials for further modification steps, especially when the goal is to obtain a composite that matches the patient’s natural enamel shades.

**Figure 4:**
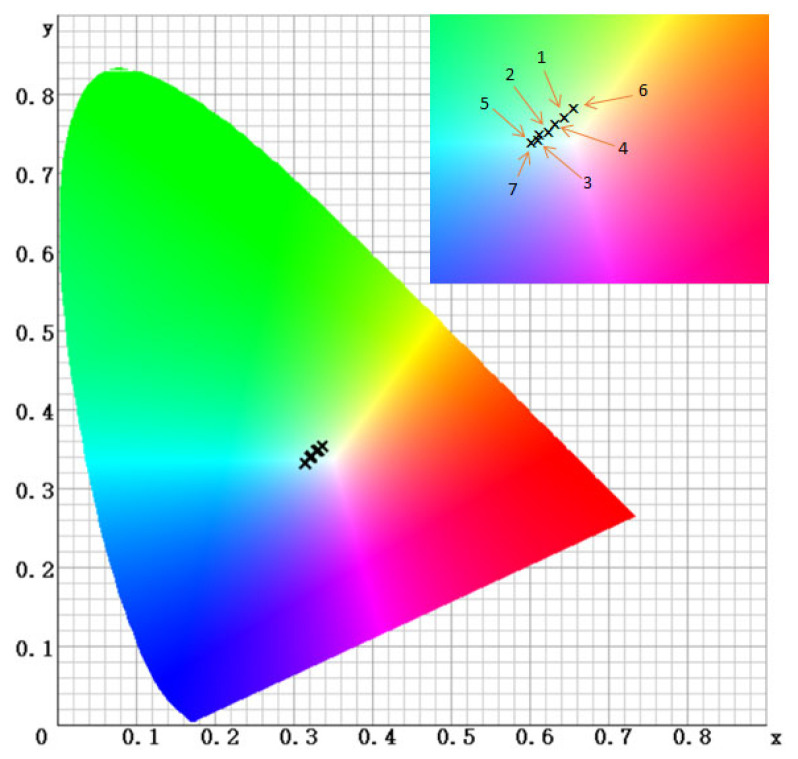
Color coordinate xy of CIE diagram for tested composites: 1—DQ1-PhTAA, 2—DQ2-PhTAA, 3—DQ3-PhTAA, 4—DQ4-PhTAA, 5—DQ5-PhTAA, 6—CQ-EDMAB, 7—reference.

Figure 4 shows the xy chromaticity diagram, illustrating the color position of each sample in the hue and chroma plane (CIE 1931 model). Points numbered 1–6 correspond to composites containing different photoinitiators (DQ1–DQ5 and CQ), while 7 is the reference.

Analysis of the xy chromaticity coordinates in the CIE 1931 plane for the tested composites reveals a distinct grouping of samples, depending on the type of photoinitiator. Samples 1–5 (DQ1–DQ5) are characterized by a slightly yellowish color with relatively low saturation. On Figure 4, the corresponding points are shifted toward the red-green direction but are located closer to the center of the diagram. In contrast, the composite containing camphorquinone (sample 6) takes on a distinctly more saturated yellow hue. On the chromaticity diagram, point 6 is shifted toward more saturated colors, toward the yellow-red region, which corresponds to the warmer and more intensely colored composite. This suggests that the presence of CQ in the composite imparts a distinct yellow color to the dental restoration, while the use of DQ photoinitiators results in a significantly less saturated, subtle light-yellow hue.

Therefore, the color intensity of the composite depends, among others, on the photoinitiator color. The more intensely colored the initiator, the more intense the color of the resulting composite will be. Conversely, a weaker or colorless initiator allows for a composite with a color closer to the reference point (neutral). When selecting an initiator, which is a component of the organic matrix, it is important to consider its impact on the final color of the resulting restoration. CQ imparts a distinct yellow tint to restorations, while the new DQ derivatives allow for a lighter, more natural tone. In practice, this means that the most aesthetic restorations are obtained using photoinitiators with weaker color, so as not to adversely affect the final shade of the composite.

**Figure 5:**
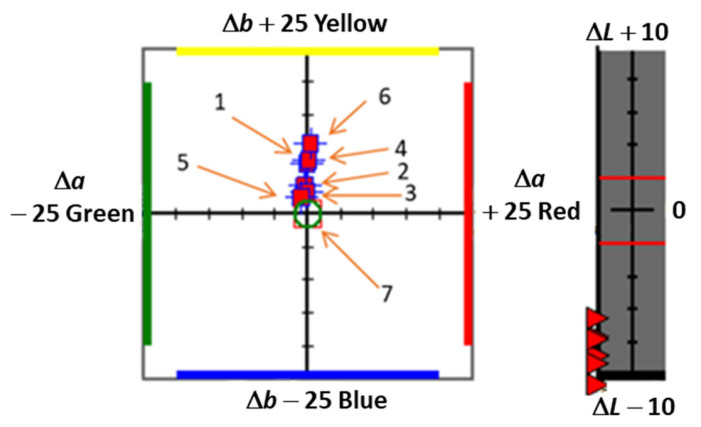
Color difference graph *L*, *a*, and *b* for tested composites: 1—DQ1-PhTAA, 2—DQ2-PhTAA, 3—DQ3-PhTAA, 4—DQ4-PhTAA, 5—DQ5-PhTAA, 6—CQ-EDMAB, 7—reference sample. The red triangles represent the lightness difference (Δ*L*) between the test color and the reference color. They indicate how much the sample differs from the reference color in lightness.

The graph of color differences Δ*L*, Δ*a*, and Δ*b* relative to the reference shows subtle but significant deviations for all composites (Figure 5).

Composites containing the DQ3-PhTAA and DQ5-PhTAA photoinitiating systems exhibited the highest Δ*L* values, meaning they were lighter than the reference sample. Sample 6 was the darkest, with the significantly lowest Δ*L* value. For most samples, Δ*a* values were negative, indicating a color shift toward green, with the largest deviation in sample DQ2-PhTAA. Only the CQ sample showed a positive Δ*a*, corresponding to a subtle reddish-yellow undertone. All tested samples exhibited positive Δ*b* values, confirming that each of the photoinitiators used imparts a warmer, yellowish hue to the composites compared to the reference material. Such detailed analysis allows us to determine not only the direction of color changes but also their intensity, which is crucial when selecting composite components with the desired brightness and tone.

### 2.2. Discoloration Resistance Tests

Stain resistance is a key quality parameter for dental materials, influencing both the aesthetics and durability of restorations. As patient expectations for the natural appearance of composite restorations grow, it is becoming increasingly important to investigate the factors that determine the materials’ tendency to discolor under the influence of external factors, such as drinks, food, or the oral environment [2,40,44].

Based on the mean values of the parameters *L*, *a*, and *b* before conditioning (0 days) and after 14 and 28 days of exposure to the tested solutions (S1–S8), the color change of the tested composites (Δ*E*) was calculated (Table 3). The Δ*E* results obtained for samples that did not exceed the threshold of clinically acceptable color change (ΔE > 3.3) are marked in red.

**Figure 6:**
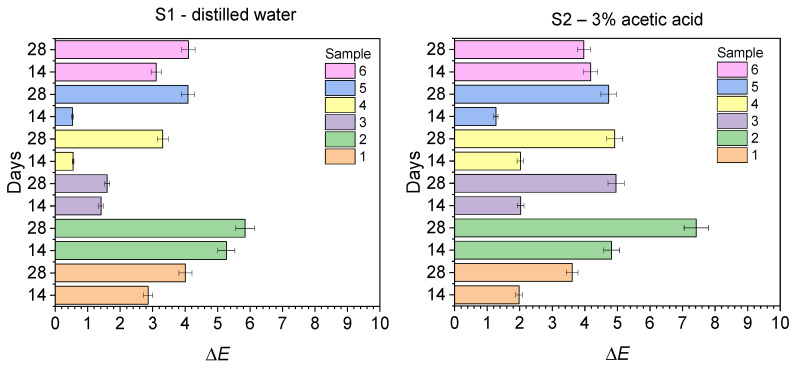

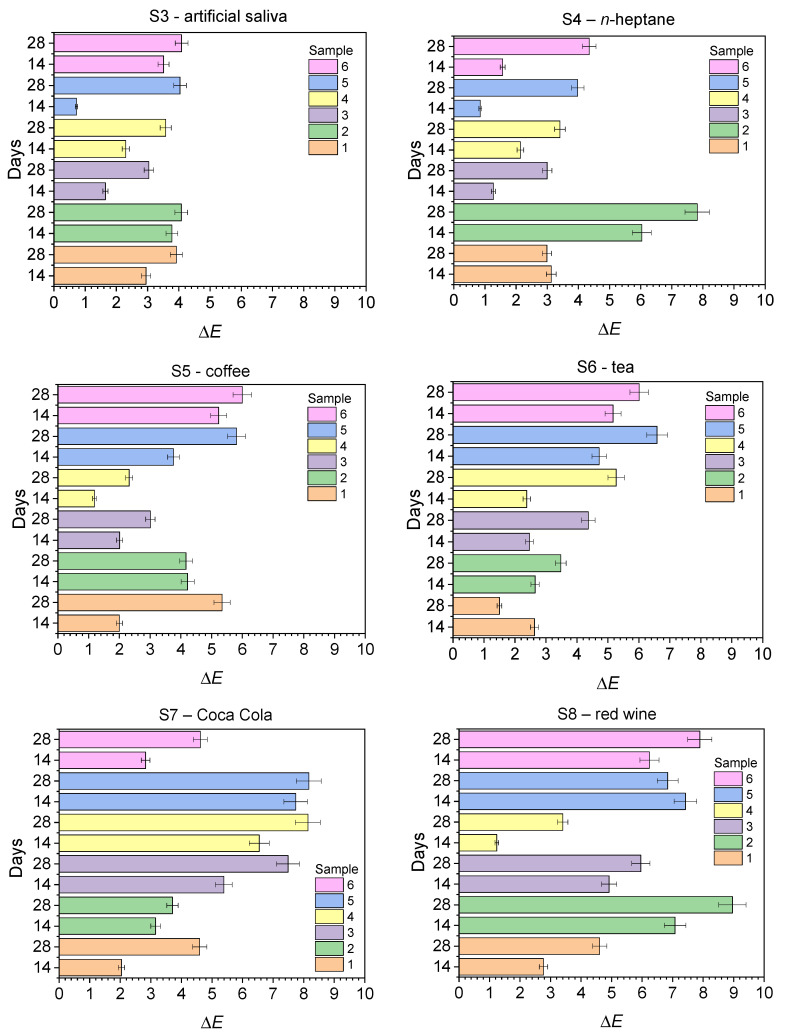
Dependence of the color change (Δ*E*) of the tested composites on the conditioning time: 1—DQ1-PhTAA, 2—DQ2-PhTAA, 3—DQ3-PhTAA, 4—DQ4-PhTAA, 5—DQ5-PhTAA, 6—CQ-EDMAB.

Figure 6 shows the dependence of the color change of the tested composites (Δ*E*) after 14 and 28 days of conditioning in the solutions used (S1–S8).

Analysis of the data in Table 2 and Figure 6 confirms that in most of the analyzed samples, statistically significant color differences compared to the initial color coincide with the point where the clinically acceptable Δ*E* limit (>3.3) is exceeded. The smallest color changes were observed in artificial saliva, distilled water, and *n*-heptane for composites 3 and 5. Only sample 6, after 14 days of storage in Coca-Cola, showed a Δ*E* lower than for artificial saliva and distilled water. In the case of samples 1 and 2, the discoloration was the smallest in tea. The average color difference in artificial saliva and distilled water for all tested composites except sample 2, even after 28 days, did not significantly exceed the Δ*E* ≈ 4.0 threshold, which in practice translates into relatively minor discoloration. Similar color changes were also observed when the sample was conditioned in a solution simulating an oily, hydrophobic environment—*n*-heptane. This likely results from the significant hydrophilicity of the tested materials and is consistent with the lowest *n*-heptane sorption reported in our previous studies [39]. Moderate discoloration was observed in 3% acetic acid solution except for sample 2 (Δ*E* = 7.42 after 28 days). Acetic acid, as a salivary demineralizing agent, causes dissolution of the composite matrix and significant weight loss, thus affecting the initial component ratio, which may further deepen the discoloration.

However, the greatest color changes in the tested composites occurred in solutions containing strong pigments, such as Coca-Cola and red wine. After 14 days of conditioning of composites 3, 4, and 5 in Coca-Cola and 2, 5, and 6 in red wine, the color difference was significant (Δ*E* > 5.0), and after 28 days, it exceeded 7.0, which corresponds to clinically significant discoloration. This is due to the high hydrophilicity of the matrix, which results in the sorption of solutions containing pigments, which in turn leads to discoloration. In most cases, exceeding permissible thresholds usually occur after 14 days of conditioning. Further extension of exposure time leads to further increases in Δ*E* and decreased color stability.

Samples 5 and 3 were found to be the most stable to discoloration after conditioning in artificial saliva, distilled water, 3% acetic acid, and *n*-heptane. Samples 1 and 4, on the other hand, showed greater color stability in coffee, tea, Coca-Cola, and red wine. In these cases, the Δ*E* remains around 5.0 even after 28 days, indicating good stain resistance. Samples 2, 5, and 6 show moderate stability in red wine, with Δ*E* significantly exceeding 5.0. Sample 4 showed the lowest resistance to discoloration in Coca-Cola, with the highest Δ*E* values observed after both 14 and 28 days of conditioning. Sample 5 is particularly susceptible to discoloration caused by dyes contained in red wine and Coca-Cola.

To assess the clinical acceptability of the tested dental composites, Δ*E* values were converted to NBS units using Formula (2) (Table 4).

The tested samples generally exhibit an increase in color change with increasing conditioning time from 14 to 28 days. The smallest increases in NBS units (i.e., the smallest color change) were observed in the environments most similar to natural ones—distilled water (S1) and artificial saliva (S3). After 14 days, the NBS units for these media range from 0.48 to 3.22 for all samples except 2, corresponding to trace or noticeable changes. After 28 days, these values increase to a maximum of 5.38 (noticeable–significant). In the acidic environment of a 3% acetic acid solution (S2), the changes are more pronounced: after 14 days, some samples (e.g., samples 2 and 6) reach values exceeding 3.8 NBS (significant change), and after 28 days, sample 2 exceeds the threshold of 6.8 NBS, which is classified as a significant color change. Similarly, in the *n*-heptane (S4), sample 2 reaches over 7.1 NBS (large) after 28 days of conditioning, while most of the other samples maintain a change in the range of 2–4 NBS (noticeable–significant).

Among the solutions containing dyes, Coca-Cola (S7) and red wine (S8) have the strongest effect on discoloration. After 14 days, several samples in both media exceed the 4–6 NBS threshold (significant–large change), and after 28 days, the highest values were recorded for samples 4 and 5 in S7 (7.48–7.51 NBS) and for sample 2 in S8 (8.24 NBS), indicating a large and clearly perceptible color change.

In terms of the stability of individual composites, sample 3 was the most resistant to change in distilled water (S1), artificial saliva (S3), and *n*-heptane (S4). NBS values remained ≤1.5 after both 14 and 28 days of storage, indicating its high resistance under neutral conditions. However, it should be noted that in strong staining environments, such as red wine (S8) or Cola-Cola (S7), the stability of this sample was significantly lower, reaching significant or large values after 28 days. Sample 5 also demonstrated good resistance to neutral environments, particularly in distilled water and artificial saliva, where initial color changes were minimal (0.48 and 0.66 NBS units, respectively). However, its susceptibility to discoloration in coffee (S5), wine (S8), and Coca-Cola (S7) was significant, reaching values above 7 NBS units, indicating high sensitivity to strongly staining media. Sample 4 exhibited exceptionally low discoloration in wine after 14 days (1.14 NBS), but was less stable in other environments, particularly Coca-Cola. Sample 2, on the other hand, proved the least resistant to color changes. In most environments, such as 3% acetic acid (S2), *n*-heptane (S4), and red wine (S8), it exhibited high NBS values (often above 6.0), qualifying as large discoloration.

Analysis of the influence of individual environments indicates that the least aggressive environments for color stability were distilled water (S1), artificial saliva (S3), and *n*-heptane (S4), in which most samples maintained color change values within clinically acceptable limits. In contrast, the most staining environments were coffee (S5), red wine (S8), and Coca-Cola (S7), in which the largest differences between samples and the highest NBS values were observed.

In summary, solutions containing dyes (especially red wine, Coca-Cola, coffee, and tea) and acidic solutions significantly impair the color stability of composites, while distilled water and artificial saliva promote color uniformity even after a month of conditioning. Samples 3 and 5 demonstrate the best stain resistance under all conditions tested.

### 2.3. Sorption and Solubility Studies

Sorption and solubility of dental composites are important parameters influencing the durability and stability of materials used to restore dental cavities [18,28,40,45]. Both phenomena are directly related to the penetration and retention of liquid molecules within the composite structure. Their effectiveness depends on the chemical properties of the polymer matrix, the degree of cross-linking, and the content of the inorganic phase [46]. Excessive water sorption can lead to swelling, plasticization of the material, and mechanical degradation, while solubility influences mass loss due to the leaching of low-molecular-weight components, which promotes the formation of micro-cavities and changes in surface properties [19,21,47,48].

According to the ISO 4049 standard [49], these parameters should be assessed quantitatively because they have a direct impact on the durability of dental fillings in the oral environment. Oral conditions—including variable pH, temperature, and the presence of saliva and staining fluids—can accelerate sorption and solubility processes. Therefore, their assessment is an important step in the design of new materials with enhanced chemical and aesthetic stability.

Table 5 presents the calculated water sorption (*S_p_*) and water solubility (*S_l_*) values for the tested composites after 14 and 28 days of conditioning. A significant increase in water sorption was observed in all tested composites with increasing conditioning time from 14 to 28 days. The highest *S_p_* sorption values were recorded for DQ2-PhTAA (sample 2), reaching 22.92 ± 1.30 mg/mm^3^ after 14 days and 25.47 ± 1.03 mg/mm^3^ after 28 days, which indicates the relatively high hydrophilicity of the matrix. In turn, the DQ5-PhTAA sample (sample 5) was characterized by the lowest sorption, 1.27 ± 1.19 mg/mm^3^ and 3.39 ± 2.23 mg/mm^3^ after 14 and 28 days, respectively, which suggests a greater resistance to water penetration in the structure of the polymerized sample.

**Figure 7:**
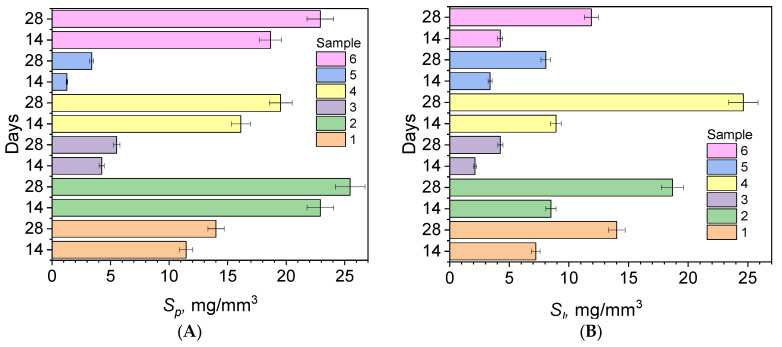
(**A**) Dependence of the average sorption changes and (**B**) dependence of the average solubility change of the tested composites on the conditioning time in distilled water: 1—DQ1-PhTAA, 2—DQ2-PhTAA, 3—DQ3-PhTAA, 4—DQ4-PhTAA, 5 –DQ5-PhTAA, 6—CQ-EDMAB.

Figure 7 illustrates the changes in the mean *S_p_* and *S_l_* values over time. An increasing trend is visible for each composite, with samples 2 and 6 demonstrating the highest sorption capacity. On the other hand, sample 5 shows a low increase in sorption even after 28 days of conditioning in distilled water. Low standard deviations indicate good repeatability of the measurements. Furthermore, a significant increase in solubility (*S_l_*) was observed for all composites with increasing conditioning time in distilled water. The largest increase in solubility was observed for sample 4 (DQ41-PhTAA), for which *S_l_* reached 24.63 ± 0.95 mg/mm^3^ after 28 days. Sample 3 (DQ3-PhTAA) showed the lowest solubility—only 4.25 ± 0.23 mg/mm^3^ after 28 days. These differences are likely due to the different degrees of cross-linking and hydrophilic properties of the polymer matrix.

A relationship between color change (Δ*E*) and sorption and solubility was also observed. Composites with the highest water sorption (*S_p_*) and highest solubility (*S_l_*) simultaneously exhibited the most significant color changes (Δ*E*) during conditioning in distilled water and dye solutions. In particular, samples DQ2-PhTAA (2), DQ4-PhTAA (4), and CQ-EDMAB (6) were characterized by high *S_p_* and *S_l_* values (ca. 20 mg/mm^3^ after 28 days) and corresponding high color change (Δ*E* > 6), indicating that their hydrophilic matrix facilitates both water absorption and dye penetration. Samples DQ3-PhTAA (3) and DQ5-PhTAA (5) showed the lowest sorption and solubility, which correlated with a mild increase in Δ*E* (below 5 in most conditions). Due to their low water and dye absorption capacity, these materials have improved color stability, making them promising candidates for applications requiring long-lasting aesthetics.

The obtained data indicate a dual mechanism for discoloration: on the one hand, water sorption leads to matrix expansion and opening diffusion channels for dyes; on the other hand, solubility causes the leaching of the components and the formation of surface defects that favor their adsorption. Selecting materials with low sorption and solubility can significantly improve the aesthetic durability of restorations, especially in environments rich in staining fluids such as coffee, tea, or red wine. In summary, the simultaneous analysis of sorption, solubility and color change allows for a comprehensive assessment of the long-term aesthetics of dental composites.

Moreover, the obtained results are consistent with the observations described in the literature regarding composite materials based on traditional photoinitiators such as camphorquinone (CQ) and matrices containing Bis-GMA or UDMA monomers. The results show that the reference composite with the CQ-EDMAB system shows significantly higher color change values (Δ*E*), especially after exposure to solutions containing strong pigments, such as red wine or Coca-Cola. Δ*E* values exceeding 6–7 are consistent with the results of previous studies, in which CQ-based composites also showed Δ*E* above the clinically acceptable threshold after 28 days of conditioning in colored solutions [50]. Bagheri et al. [50] showed that light-cured composites containing CQ changed color by more than 7 Δ*E* units after a few weeks of soaking in wine and tea, which corresponds to a noticeable and clinically significant shade change. Ferracane [2] and Sideridou [19] also reported the susceptibility of Bis-GMA and UDMA-based composites to discoloration and dye absorption in the oral environment.

In terms of sorption and solubility, the CQ-containing sample was characterized by significantly higher water absorption and higher solubility compared to the experimental materials containing quinoline and quinoxaline derivatives (DQ1-DQ5). The exception was sample 2, which showed higher water sorption than sample 6. However only, the solubility values for samples 3 and 5 were lower than for the reference, regardless of the conditioning time.

These results are consistent with the reports of Ferracane et al. [18], who demonstrated that conventional Bis-GMA-based matrices are characterized by relatively high hydrophilicity and increased susceptibility to sorption, which in turn correlates with greater material degradation. The sorption value of the reference composite (over 22 mg/mm^3^) is comparable to the results of Sideridou and Karabeli [19], in which nanohybrid materials achieved sorption levels in the range of 15–25 mg/mm^3^.

Composites with the newly developed DQ3-PhTAA and DQ5-PhTAA systems stand out favorably, demonstrating both lower Δ*E* values (<8) and significantly lower susceptibility to sorption (<6 mg/mm^3^) and solubility (<8 mg/mm^3^). Their color stability and chemical resistance demonstrate the potential for these materials in applications requiring high aesthetics and long-term durability. The obtained data confirm that reducing matrix hydrophilicity and using photoinitiators with favorable optical properties can significantly improve the performance of composite materials, providing an alternative to traditional photoinitiator systems.

## 3. Materials and Methods

### 3.1. Reagents

The synthesis of photoinitiators DQ1-DQ5 is described in our previous papers [38,39]. The remaining components of the organic matrix of the dental composites were purchased from Sigma-Aldrich Co. (St. Louis, MO, USA). The filler constituting the inorganic phase—neutral dental glass—IDG (Inter Dental Glass) GM 35429—was purchased from Schott Dental Glass Co. (Wolverhampton, UK). Reagents simulating the natural oral environment during consumption of various meals were purchased from the companies mentioned in our previous work [39].

### 3.2. Methods

#### 3.2.1. Sample Preparation

For this study, 162 cylindrical samples simulating dental fillings were prepared. 144 samples were used to evaluate color stability, and 18 were used for sorption and solubility analysis.

Appropriate amounts of photoinitiator (DQ1-DQ5) and electron donor (PhTAA) were dissolved in 0.1 mL of 1-methyl-2-pyrrolidone (MP). The concentrations of photoinitiators ranged from 1.35 × 10^−3^ to 1.80 × 10^−3^ M depending on the molar absorption coefficient [38], and the co-initiator concentration was 0.1 M. Then, 0.9 mL of monomer (TMPTA) was added. The organic matrix was thoroughly mixed in a mortar with 1.50 g of inorganic IDG filler. The composites were photocured using a dental LED lamp (Cromalux 75 Mega Physik Dental, Rastatt, Germany) with an emission range of 390–500 nm and an intensity of 20 mW/cm^2^ in a Teflon ring. Irradiation was continued for approximately 60 s until the sample was completely polymerized. For each of the six photoinitiator-coinitiator systems, 27 cylindrical samples were prepared with dimensions of 10 mm diameter × 3 mm height.

The properties of the experimental dental composites were verified by comparing the tested systems with samples containing commercial compounds. The liquid organic matrix contained compounds commonly used in dentistry, such as a CQ (photoinitiator), an EDMAB (co-initiator), and a Bis-GMA (monomer). The concentration of camphorquinone was 0.675 M due to its low molar absorption coefficient (40 M^−1^ cm^−1^).

To obtain real color change of the tested photoinitiators, the prepared composite materials, apart from the reagents given in Table 6, did not contain any auxiliary substances such as light stabilizers, compounds allowing selection of the appropriate shade of the filling to the natural color of the patient’s teeth, or inhibitors preventing premature polymerization during storage.

The composition of the composite materials tested and reference systems are presented in Table 6.

#### 3.2.2. Color Stability Testing Methodology

**Figure 8:**
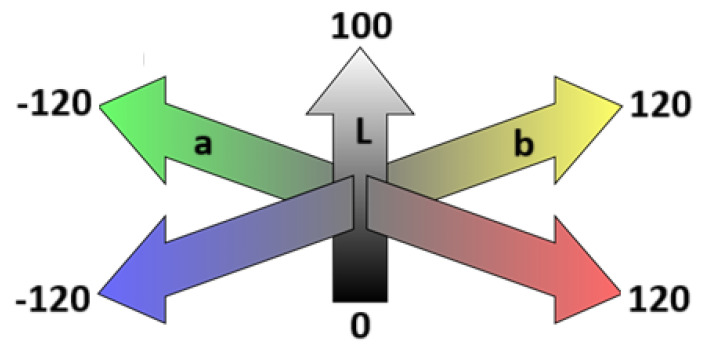
Diagram of color changes along the *L*, *a*, and *b* axes in the CIE Lab color space.

Colorimetric measurement in the CIE Lab color space, developed by the International Commission on Illumination [28,31,32,51,52], was used to assess the color stability of dental hard tissue restoration materials. The CIE Lab scale defines the numerical value of color, characterizing it using three coordinates: *L*, *a*, and *b*. The *a*-axis represents the proportion of green or red in the analyzed color, with shades of green having a negative value and shades of red having a positive value (*a*: from −120 to +120). The *b*-axis represents the proportion of blue or yellow in the analyzed color, with shades of blue having a negative value and shades of yellow having a positive value (*b*: from −120 to +120). The *L*-axis describes the lightness of the color, ranging from 0 (black) to 100 (white). Color changes in the CIE Lab space are shown in Figure 8.

Before measurement in the CIE Lab color space, the samples were prepared to be uniform and matte or semi-matte to minimize errors resulting from directional reflections. Immediately after the photopolymerization process, they were stored in a dark container at 23 °C and approximately 50% relative humidity for 24 h. Then, the sample surfaces were polished successively with P600, P1200, and P2000 abrasive paper, rinsed with deionized water, and air-dried.

The coordinates *L*, *a*, and *b* were measured using an SV-100/300 spectrophotometer (TRI-COLOR sp. z o. o., Narama, Poland), repeating the measurement three times (n = 3). The values Δ*L*, Δ*a*, and Δ*b* were determined as the differences between the values measured for the tested sample and the reference values (*L* = 98.93; *a* = −0.28; *b* = 0.54) obtained on a standard tooth whiteness chart [51]. The color change was determined using the parameter Δ*E* calculated based on Equation (1).

(1) $$∆E=\sqrt{∆L^{2}+∆a^{2}+∆b^{2}},$$

where Δ*L*, Δ*a*, and Δ*b* denote the changes in the values of individual coordinates between the tested sample and the reference sample [51].

According to the criteria accepted in the literature, values of Δ*E* < 1 remain almost imperceptible to the human eye, Δ*E* in the range from 1 to 3.3 is considered noticeable to a skilled observer but clinically acceptable, while Δ*E* > 3.3 is perceived by an unskilled observer and considered clinically unacceptable [53]. Some authors recommend adopting a threshold value of Δ*E* = 3.7 as the criterion for unacceptable color change [32,52,54].

Additionally, the color difference assessment was supplemented by the NBS (National Bureau of Standards) scale, which, like Δ*E*, reflects the human perception of color differences. The Δ*E* values were converted to NBS units using Equation (2).

NBS = *ΔE*·0.92, (2)

The assignment of NBS values to the degree of color difference noticeable is presented in Table 7 [32].

To investigate the effect of oral conditions on color stability, samples (n = 3) were placed for 28 days in eight different solutions: distilled water, artificial saliva, *n*-heptane, 3% acetic acid, and pigment-containing solutions: coffee, tea, red wine, and Coca-Cola. The tea and coffee solutions were prepared according to the manufacturers’ instructions (Lipton Earl Grey (Purchase, NY, USA) and Jacobs Krönung (Amsterdam, The Netherlands)). After 28 days, *L*, *a*, and *b* measurements were repeated on the same sample surface, allowing for the determination of differences in Δ*L*, Δ*a*, Δ*b*, and the resulting Δ*E* between the initial state and the state after exposure to the coloring agents. The characteristics of the solutions used are presented in Table 8.

#### 3.2.3. Sorption and Solubility Test Methodology

Sorption and solubility tests were conducted in accordance with the International Organization for Standardization standard ISO 4049 [49]. For each composite, three samples were prepared, numbered, and placed in a desiccator to dry to a constant mass (m_1_). The weighing process was repeated until the mass loss of each sample exceeded 0.1 mg within 24 h.

After achieving a constant mass, the samples were immersed in 10 mL of distilled water and stored at 37 ± 1 °C for 14 and 28 days. After each predetermined time period, the samples were surface-dried with a paper towel to remove external moisture and then weighed (m_2_). Then, the samples were dried in a desiccator at 37 °C in the presence of a drying agent (silica gel) until a constant mass was achieved (±0.1 mg), after which the samples were weighed again (m_3_). After each cycle, the samples were placed in freshly prepared solutions.

The volume of the tested samples (V) was calculated in mm^3^, assuming their height as 3 mm and radius as 5 mm. Water sorption (*S_p_*) and solubility (*S_l_*), expressed in mg/mm^3^, were calculated using the following equations:

(3) $$S_{p}=\frac{m_{2}-m_{3}}{V},$$

(4) $$S_{l}=\frac{m_{1}-m_{3}}{V}.$$

## 4. Conclusions

This article discusses the sorption and solubility of new dental composites, which are important parameters determining their long-term stability, both mechanical and aesthetic. Materials with a higher capacity for water absorption and dissolution in an aqueous environment exhibit greater susceptibility to discoloration, confirming the existence of a mechanism enabling the diffusion of dyes into the inner layers of the material. Composites with low sorption and solubility, particularly DQ3-PhTAA and DQ5-PhTAA, demonstrate improved color stability, making them more suitable for aesthetic applications. This suggests that when designing new dental composites, special attention should be paid to reducing the hydrophilicity of the matrix and increasing the inorganic phase content. The use of photoinitiators with favorable physicochemical properties can further enhance the color stability of the materials. These results make a valuable contribution to the development of modern composite materials with high aesthetics and resistance to environmental factors.

## Figures and Tables

**Table 1:** Compounds included in the tested dental compositions.

Organic Phase	
Photoinitiators:	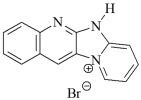 DQ1: quinoline [2,3-b]-1H-imidazo [1,2-a]pyridinium bromide
	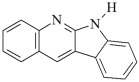 DQ2: 6H-indolo [2,3-b]quinoline
	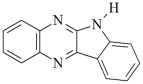 DQ3: 6H-indolo [2,3-b]quinoxaline
	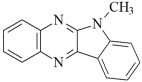 DQ4: 6-methyl-6H-indolo [2,3-b]quinoxaline
	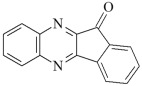 DQ5: 11H-indeno [1,2-b]qunioxalin-11-on
	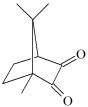 CQ: camphorquinone
Co-initiators:	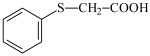 PhTAA: (phenylthio)acetic acid
	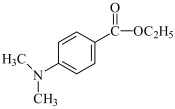 EDMAB: ethyl 4-dimethylaminobenzoate
Monomers:	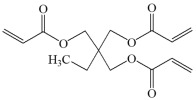 TMPTA: trimethylolpropane triacrylate
	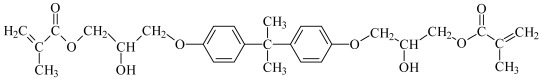 Bis-GMA: Bisphenol A glycerolate dimethacrylate
Solvent:	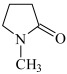 MP: 1-methyl-2-pyrolidinone
**Inorganic phase**	
Dental filler:	SiO_2_—30, CaO—10, Al_2_O_3_—30, F—15, P_2_O_5_ < 10, Na_2_O < 10% mass; ING: Inter Dental Glass

**Table 2:** Initial color difference values (Δ*E*) of the tested materials calculated based on the values of *L*, *a*, and *b* parameters in relation to the reference: 1—DQ1-PhTAA, 2—DQ2-PhTAA, 3—DQ3-PhTAA, 4—DQ4-PhTAA, 5—DQ5-PhTAA, 6—CQ-EDMAB.

Sample	1	2	3	4	5	6
Δ*E*	8.86 ± 0.56	11.72 ± 0.78	7.70 ± 0.68	12.37 ± 1.05	7.02 ± 0.34	14.57 ± 1.32

**Table 3:** The color change values (Δ*E*) of the tested materials were calculated based on the values of the *L*, *a*, and *b* parameters depending on the type of solution used and the conditioning time (the background color of the table cells shows the color of the material).

Composite Material, Δ*E*						
Storage Time, Days	1 DQ1-PhTAA	2 DQ2-PhTAA	3 DQ3-PhTAA	4 DQ4-PhTAA	5 DQ5-PhTAA	6 CQ-EDMAB
0						
S1—distilled water						
14	2.86 ± 0.15	5.27 ± 0.25	1.41 ± 0.37	0.55 ± 0.21	0.53 ± 0.13	3.11 ± 0.24
28	4.01 ± 0.23	5.85 ± 0.59	1.60 ± 0.09	3.31 ± 0.23	4.09 ± 0.67	4.10 ± 0.71
S2—3% acetic acid						
14	1.98 ± 0.09	4.82 ± 0.45	2.03 ± 0.31	2.02 ± 0.32	1.27 ± 0.20	4.18 ± 0.15
28	3.61 ± 0.35	7.42 ± 0.26	4.96 ± 0.67	4.92 ± 0.45	4.73 ± 0.37	3.97 ± 0.67
S3—artificial saliva						
14	2.95 ± 0.34	3.78 ± 0.45	1.65 ± 0.08	2.30 ± 0.13	0.72 ± 0.17	3.51 ± 0.34
28	3.92 ± 0.25	4.08 ± 0.93	3.04 ± 0.87	3.58 ± 0.45	4.04 ± 0.43	4.09 ± 0.32
S4—*n*-heptane						
14	3.13 ± 0.47	6.04 ± 0.89	1.27 ± 0.05	2.14 ± 0.87	0.85 ± 0.03	1.57 ± 0.06
28	2.99 ± 0.02	7.82 ± 0.78	3.00 ± 0.76	3.41 ± 0.34	3.98 ± 0.36	4.35 ± 0.25
S5—coffee						
14	2.00 ± 0.36	4.22 ± 0.41	2.01 ± 0.44	1.19 ± 0.56	3.76 ± 0.55	5.23 ± 0.78
28	5.34 ± 0.22	4.17 ± 0.34	3.01 ± 0.67	2.32 ± 0.33	5.81 ± 0.26	6.00 ± 0.99
S6—tea						
14	2.63 ± 0.47	2.65 ± 0.89	2.47 ± 0.21	2.38 ± 0.35	4.72 ± 0.22	5.17 ± 0.34
28	1.50 ± 0.09	3.48 ± 0.45	4.37 ± 0.56	5.27 ± 0.78	6.59 ± 0.66	6.01 ± 0.98
S7—Coca Cola						
14	2.04 ± 0.54	3.16 ± 0.78	5.39 ± 0.89	6.55 ± 0.45	7.74 ± 0.72	2.83 ± 0.09
28	4.60 ± 0.66	3.71 ± 0.55	7.49 ± 0.67	8.14 ± 0.89	8.17 ± 1.02	4.62 ± 0.30
S8—red wine						
14	2.77 ± 0.67	7.08 ± 0.77	4.92 ± 1.01	1.24 ± 0.22	7.42 ± 0.57	6.21 ± 0.98
28	4.61 ± 0.22	8.96 ± 0.54	5.96 ± 0.32	3.40 ± 0.48	6.84 ± 0.67	7.89 ± 0.84

**Table 4:** Color evaluation of the tested composites according to NBS units.

Sample	Storage Time (Days)	NBS Unit	Color Change	NBS Unit	Color Change
		S1—distilled water		S2—3% acetic acid	
1	14	2.63	noticeable	1.82	noticeable
	28	3.68	significant	3.32	significant
2	14	4.84	significant	4.43	significant
	28	5.38	significant	6.82	large
3	14	1.29	small	1.86	noticeable
	28	1.47	small	4.56	significant
4	14	0.50	slight	1.85	noticeable
	28	4.51	significant	4.52	significant
5	14	0.48	slight	1.16	small
	28	3.76	significant	4.35	significant
6	14	2.86	noticeable	3.84	significant
	28	3.77	significant	3.59	significant
		S3—artificial saliva		S4—*n*-heptane	
1	14	2.71	noticeable	2.87	noticeable
	28	3.60	significant	2.75	noticeable
2	14	3.47	significant	5.55	significant
	28	3.75	significant	7.19	large
3	14	1.51	noticeable	1.16	small
	28	2.79	noticeable	2.76	noticeable
4	14	2.11	noticeable	1.96	noticeable
	28	3.29	significant	3.13	significant
5	14	0.66	small	0.78	small
	28	3.71	significant	3.66	significant
6	14	3.22	significant	1.44	small
	28	3.76	significant	4.00	noticeable
		S5—coffee		S6—tea	
1	14	1.84	noticeable	2.41	noticeable
	28	4.91	significant	1.38	small
2	14	3.88	significant	2.41	noticeable
	28	3.83	significant	3.20	significant
3	14	1.84	noticeable	2.27	noticeable
	28	2.76	noticeable	4.02	significant
4	14	1.09	small	2.18	noticeable
	28	2.13	noticeable	4.84	significant
5	14	3.45	significant	4.34	significant
	28	5.34	significant	6.06	large
6	14	4.81	significant	4.75	significant
	28	5.52	significant	5.52	significant
		S7—Coca Cola		S8—red wine	
1	14	1.87	noticeable	2.54	noticeable
	28	4.23	significant	4.24	significant
2	14	2.90	noticeable	6.51	large
	28	3.41	significant	8.24	large
3	14	4.86	significant	4.52	significant
	28	6.89	large	5.48	significant
4	14	6.02	large	1.14	small
	28	7.48	large	3.12	significant
5	14	7.12	large	6.66	large
	28	7.51	large	6.29	large
6	14	2.60	noticeable	5.71	significant
	28	4.25	significant	7.25	large

**Table 5:** Water sorption (*S_p_*, mg/mm^3^) and water solubility (*S_l_*, mg/mm^3^) after 14 and 28 days; tested composites: 1—DQ1-PhTAA, 2—DQ2-PhTAA, 3—DQ3-PhTAA, 4—DQ4-PhTAA, 5—DQ5-PhTAA, 6—CQ-EDMAB.

Sample	*S_p_* (mg/mm^3^) Mean ± SD	*S_l_* (mg/mm^3^) Mean ± SD	*S_p_* (mg/mm^3^) Mean ± SD	*S_l_* (mg/mm^3^) Mean ± SD
	Day 14		Day 28	
1	11.46 ± 2.24	7.22 ± 0.20	14.01 ± 1.64	14.01 ± 0.74
2	22.92 ± 1.30	8.49 ± 0.31	25.47 ± 1.03	18.68 ± 0.93
3	4.24 ± 1.08	2.12 ± 0.08	5.52 ± 0.93	4.25 ± 0.23
4	16.13 ± 0.92	8.91 ± 0.32	19.53 ± 0.99	24.63 ± 0.95
5	1.27 ± 1.19	3.39 ± 0.21	3.39 ± 2.23	8.06 ± 0.26
6	18.68 ± 1.05	4.24 ± 0.35	22.92 ± 0.98	11.88 ± 0.48

**Table 6:** Dental composites used for the tests; photoinitiator concentration ranged from 1.38 × 10^−3^ to 0.675 M (given in the table), the co-initiator concentration was 0.1 M, the TMPTA-MP ratio was 9:1 *v*/*v*, and the inorganic phase was 60% of the organic phase.

No.			c, M	Organic Phase				Inorganic Phase
				Photoinitiator	Co-Initiator	Monomer	Solvent	
1	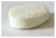	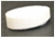	1.80 × 10^−3^	DQ1	PhTAA	TMPTA	MP	IDG
2	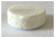	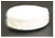	1.35 × 10^−3^	DQ2	PhTAA	TMPTA	MP	IDG
3	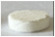	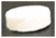	1.38 × 10^−3^	DQ3	PhTAA	TMPTA	MP	IDG
4	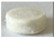	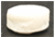	1.58 × 10^−3^	DQ4	PhTAA	TMPTA	MP	IDG
5	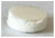	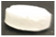	1.54 × 10^−3^	DQ5	PhTAA	TMPTA	MP	IDG
6	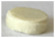	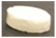	0.675	CQ	EDMAB	Bis-GMA	MP	IDG

**Table 7:** Color grading scale according to NBS units [32].

NBS Units	Color Change
0.0–0.5	slight
0.5–1.5	small
1.5–3.0	noticeable
3.0–6.0	significant
6.0–12.0	large
≥12	very large

**Table 8:** Characteristics of solutions used in color stability tests of the studied composites.

No	Solution	Simulated Environment	
S1	distilled water	hydrated foods	
S2	3% acetic acid	hydrated foods with pH < 4.5	
S3	artificial saliva	saliva	
S4	*n*-heptane	fatty foods	
S5	coffee	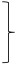	foods containing pigments
S6	tea		
S7	Coca-Cola		
S8	red wine		

## Data Availability

All data generated or analyzed during this study are included in this published article.
